# Financial indicators utilization among community pharmacists: A comprehensive study for pharmacy management

**DOI:** 10.1371/journal.pone.0299798

**Published:** 2024-03-01

**Authors:** Mohammad Abu Assab, Hisham E. Hasan, Hamza Alhamad, Fares Albahar, Abdallah Alzayadneh, Hanadi Abu Assab, Wael Abu Dayyeh, Zainab Zakaraya

**Affiliations:** 1 Department of Clinical Pharmacy, Faculty of Pharmacy, Zarqa University, Zarqa, Jordan; 2 Department of Pharmacy, Faculty of Pharmacy, Mutah University, Al-Karak, Jordan; 3 Biopharmaceutics and Clinical Pharmacy Department, Faculty of Pharmacy, AL-Ahliyya Amman University, Amman, Jordan; Isra University Faculty of Pharmacy, JORDAN

## Abstract

**Background:**

The financial management of community pharmacies is a critical aspect of healthcare delivery, as pharmacists often operate as healthcare providers and business managers. Understanding pharmacists’ awareness, perceptions, and practices related to financial indicators is essential for effective pharmacy management. There is a paucity of research addressing this issue regionally and locally.

**Objectives:**

This study aimed to investigate the perceptions and utilization of financial indicators among community pharmacists in Jordan and identify demographic and contextual factors influencing their financial practices.

**Methods:**

A cross-sectional study was conducted, surveying 353 community pharmacists from various regions of Jordan. The developed and validated survey assessed demographic characteristics, utilizations of financial indicators, and perceptions of their significance. Pharmacists were queried about their financial practices, including the use of various financial indicators. Descriptive and analytical statistics were used to portray the study’s findings.

**Results:**

The study included a diverse group of community pharmacists in terms of demographic characteristics. Most pharmacists exhibited awareness of financial indicators, with a higher awareness of profitability and liquidity indicators. Pharmacists generally had positive perceptions of the importance of these indicators in daily practice. High agreement was observed in financial practices, including following up on payables and receivables, monitoring changes in monthly revenue, and preparing income statements. There was significant variation in the utilization and perception of financial indicators based on factors such as pharmacy ownership, province, foundation age, and practical experience.

**Conclusion:**

The findings indicate a positive correlation between utilization and perception, emphasizing the importance of raising awareness of financial indicators among pharmacists. The study also highlights the significance of tailored financial training programs for pharmacists at different stages of their careers and the importance of regional context in financial practices. Understanding these variations can lead to more effective financial management and improved healthcare services in community pharmacies.

## Introduction

In the evolving landscape of community pharmacy practice, financial management has emerged as a critical area that demands increased attention [1]. As community pharmacists in Jordan continue to be at the forefront of healthcare service delivery, it is essential to recognize that their roles extend beyond the traditional realms of medication dispensing and counseling [2–4]. In today’s complex and dynamic healthcare environment, pharmacists are healthcare entrepreneurs who must navigate economic challenges and harness financial indicators to thrive [5–7].

Influenced by demographic shifts, technological advancements, and changing patient expectations, the global healthcare ecosystem has transformed in recent years [8–10]. Community pharmacies have evolved into comprehensive healthcare centers, offering a range of services that encompass disease management, wellness programs, and preventive care. This expansion of services is a response to the rising demand for accessible and convenient healthcare solutions [11–13]. Consequently, the financial dynamics of community pharmacies have grown in complexity [14].

Moreover, the ongoing COVID-19 pandemic has accelerated the adoption of telehealth services, digital health tools, and remote patient monitoring [15, 16]. Community pharmacists have embraced these technologies to engage with patients, expand their reach, and diversify their sources of income [17]. These innovations have changed the financial landscape, necessitating a deeper understanding of financial indicators tailored to modern community pharmacy [18, 19].

In this era of data-driven decision-making, community pharmacists face the challenge of harnessing the power of financial indicators to optimize their operations, enhance patient care, and secure their financial sustainability [20–22]. These indicators offer insights into liquidity management, profitability assessment, leverage optimization, and operational efficiency, providing a comprehensive view of a pharmacy’s financial health [23].

Financial indicators encompass four primary dimensions: liquidity, profitability, leverage (solvency), and operations. Liquidity indicators assess an organization’s ability to meet short-term obligations by converting current assets into cash, encompassing elements such as inventory, receivables, and short-term investments. Profitability indicators gauge a pharmacy’s capacity to generate profits from its resources. Leverage ratios delve into the sources of asset funding, particularly long-term debt. Finally, operations ratios, specific to each business, analyze efficiency and effectiveness [24].

The economic responsibilities of community pharmacies form the foundation of corporate social responsibilities, as outlined in Carroll’s four-level model: economic, legal, ethical, and philanthropic [25]. The economic dimension underscores the priority of profit generation and economic growth. It is imperative for community pharmacies to improve their financial performance and operational efficiency in order to ensure their financial health.

Against this backdrop of transformative trends and evolving roles, this study seeks to explore the significance of using financial indicators among community pharmacists in Jordan. There is a scarcity of research addressing this issue within the Jordanian context, both regionally and locally. By delving into the nuances of financial management in the context of contemporary pharmacy practice, we aim to provide valuable insights that can inform education and training programs.

As community pharmacists in Jordan continue to adapt and innovate, their ability to grasp the intricacies of financial indicators will be pivotal in shaping the future of pharmacy practice. This study contributes to the ongoing dialogue on community pharmacy’s financial management trends and equips pharmacists with the knowledge and skills needed to excel in this dynamic healthcare landscape.

## Materials and methods

### Study design and participants

This research employed a cross-sectional study design to investigate financial indicators’ significance among Jordan’s community pharmacists. The study’s target population consisted of licensed community pharmacists practicing in various regions of Jordan. Pharmacists of different ages, experience levels, and ownership statuses were included in the study to ensure a diverse and representative sample.

### The questionnaire tool

The questionnaire was developed based on the study objectives, a thorough literature review [14, 26–28], and consultation with pharmacy management and finance experts. Drafted in simple Arabic, the questionnaire aimed to elicit clear and unambiguous responses from a diverse group of pharmacists. Combining both closed-ended and five-point Likert-scale questions to gather quantitative data. It was pre-tested with a small sample of pharmacists to ensure clarity, relevance, and reliability. Respondents’ feedback was incorporated to improve the questionnaire’s quality and validity. The final draft consisted of two parts: the first focused on gathering demographic information about pharmacists (gender, age, qualification, experience, and ownership status) and their pharmacies (location, type, and foundation age), while the second explored the most relevant financial indicators that community pharmacists utilize, such as liquidity ratios, profitability measures, leverage ratios, and operational efficiency metrics, in their daily practice and decision-making processes, focusing on implementing financial analysis in pharmacy practice.

### Data collection

Data collection was conducted using the structured questionnaire designed to assess participants’ awareness and perception of financial indicators over four months (from January to April 2023). An online survey platform was utilized to distribute the questionnaire electronically. Pharmacists were invited to participate through social media groups. This method allowed for the inclusion of pharmacists from remote areas and ensured a broader geographical representation. Trained research assistants visited some community pharmacies in Jordan using convenience sampling techniques to administer the questionnaire in person. This approach was particularly useful for reaching pharmacists who preferred face-to-face interactions or had limited access to online resources.

### Ethical considerations

This study adhered to ethical principles and obtained ethical approval from the institutional review board (IRB) of the Scientific Research Ethics Committee at Zarqa University (approval number: 1/2/2022). All participants provided informed consent before participating in the study. Confidentiality and data security were maintained throughout the research process, and participants’ identities were anonymized to ensure their privacy and protection.

### Data analysis

The data obtained from the surveys was analyzed using the Statistical Package for Social Science (SPSS) software, version 23. Descriptive statistics, including frequencies and percentages, were used to summarize participants’ demographic characteristics and responses to perception questions. Inferential statistics such as *chi-*squared tests, one-sample *t-*tests, and analysis of variance (ANOVA) were applied to identify potential differences in perception among different subgroups (e.g., age groups, ownership status). Questionnaire consistency and reliability were measured using Cronbach’s α test, and the overall internal consistency of the study was sufficient (*α* = 0.96), within the commonly accepted range of 0.45–0.96 in social science research [29]. A significance level of *p* < 0.05 was set to determine statistical significance in all tests.

## Results

### Demographic characteristics of participants

A total of 353 community pharmacists from various regions of Jordan participated in the study. Table 1 presents an overview of the demographic characteristics of the participants. The study participants represented a diverse group of community pharmacists. Most participants were female (53.5%); aged 45 years or older (43.3%); had a BSc degree in pharmacy (83.0%); worked in independent pharmacies (91.8%) in the central region of Jordan (66.0%); most of them were owners (78.8%); and had ten years or more of working experience in community pharmacies (56.7%) with more than ten years of establishment (46.5%). Regarding the particular question about obtaining financial performance information (indicators of profitability, liquidity, leverage, and efficiency) for the pharmacy, most pharmacists (64.6%) used self-effort, according to their experience and knowledge.

**Table 1:** Demographic characteristics of study participants (N = 353).

Variables	Categories	N (%)
**Gender**	Male	164 (46.5)
	Female	189 (53.5)
**Pharmacist Age Group**	25–34 years	116 (32.9)
	35–44 years	94 (23.9)
	45 years or older	153 (43.3)
**Qualification**	Doctor of Pharmacy (PharmD)	18 (5.1)
	Bachelor of Pharmacy (BSc)	293 (83.0)
	Postgraduate Studies	42 (11.9)
**Geographical Location**	Northern Region	86 (24.4)
	Central Region	233 (66.0)
	Southern Region	34 (9.6)
**Ownership Status**	Single Pharmacy Owner	324 (91.8)
	Chain Pharmacy Owner	29 (8.2)
**Pharmacy’s Foundation Age**	Less than five years	103 (24.3)
	Five to Nine years	86 (29.2)
	Ten years or older	164 (46.5)
**Pharmacist Financial Authorization**	Owner	278 (78.8)
	Authorized	75 (21.2)
**Years of Experience**	Less than five years	76 (21.5)
	Five to Nine years	77 (21.8)
	Ten years or older	200 (56.7)
**Obtaining the financial performance information (indicators of profitability, liquidity, leverage, and efficiency) for the pharmacy through**	Accounting and financial company	14 (4.0)
	a pharmacy accounting program	1 (0.3)
	Do not evaluate the financial performance of the pharmacy	30 (8.5)
	Self-effort, according to experience and knowledge	228 (64.6)
	Hiring a professional accountant	71 (20.1)
	Pharmacy’s financial manager	3 (0.8)
	Relying on the final net profit only	1 (0.3)
	There is an accountant at the end of each month	4 (1.1)
	Based on a system	1 (0.3)

### Perception and practice of financial indicators

Table 2 presents pharmacists’ financial indicators, practices, and activities performed within their daily pharmacy practice. The majority of pharmacists (74.22%) agreed with the practice of following up on payables and receivables periodically, indicating a high level of caution in managing financial obligations with a mean score of 4.05 ± 1.07 and a high significance test (*t* = 18.42, *p* < 0.001). More than two-thirds of pharmacists (69.12%) indicated their involvement in monitoring changes in monthly revenue, demonstrating their commitment to financial oversight with a mean score of 3.81 ± 1.06 with high significance (*t* = 14.37, *p* < 0.001). Over half of the pharmacists (58.07%) reported the preparation of income statements, reflecting a key financial reporting activity with a mean score of 3.63 ± 1.14 (*t* = 10.38, *p* < 0.001). The total mean score for all responses was 3.50 ± 0.92, further affirming the significance of these financial practices in the pharmacy setting with statistical significance (*t* = 10.31, *p* <0.001).

**Table 2:** Pharmacists’ financial indicators practices and activities performed (N = 353).

No.	Variables Statements	Agreed and strongly agreed	Sample’s mean	Mean significance	
		N (%)	M ± SD	*t*	*p-*value
**1**	We follow up on payables and receivables periodically.	262 (74.22)	4.05 ± 1.07	18.42	<0.001
**2**	We calculate the amount of change in monthly revenue	244 (69.12)	3.81 ± 1.06	14.37	<0.001
**3**	We prepare the income statement.	205 (58.07)	3.63 ± 1.14	10.38	<0.001
**4**	We divide the net profit by the total revenue to find out the pharmacy’s profit margins	201 (56.94)	3.52 ± 1.19	8.16	<0.001
**5**	We prepare the balance sheet.	192 (54.39)	3.52 ± 1.22	7.97	<0.001
**6**	We prepare the trial balance at the end of the financial period.	185 (52.41)	3.45 ± 1.20	7.12	<0.001
**7**	We do a financial analysis of the pharmacy periodically.	184 (52.12)	3.56 ± 1.03	10.19	<0.001
**8**	We prepare a cash flow statement.	183 (51.84)	3.44 ± 1.10	7.62	<0.001
**9**	We use computer programs to generate financial statements.	177 (50.14)	3.28 ± 1.29	4.03	<0.001
**10**	We compare the primary objectives of the balance sheet and income statement.	168 (47.59)	3.29 ± 1.15	4.72	<0.001
**11**	We create financial statements using Excel	166 (47.03)	3.24 ± 1.31	3.37	<0.001
**12**	We make a relationship between the balance sheet and the income statement for a particular financial year.	157 (44.48)	3.25 ± 1.17	3.97	<0.001
**Total**		2324 (54.86)	3.50 ± 0.92	10.31	<0.001

Table 3 presents the analysis of pharmacists’ financial performance indicators currently applied in their practice, which includes various financial performance metrics categorized into four dimensions. The first category comprises metrics that evaluate the financial profitability of pharmacists. The majority agreed on utilizing profitability items, with 747 participants (52.90%) indicating their agreement with a mean score of 3.44 ± 0.96 (*p* < 0.001). Notably, the highest score was observed for "Profit Margin" (67.42%). The second category, liquidity, examines the ability of pharmacists to meet their short-term financial obligations. In this category, 258 participants (36.54%) agreed with using it with a mean score of 3.05 ± 1.15, with all indicators showing non-significant differences (*p* > 0.05). The leverage category assesses the extent to which pharmacists use debt financing in their practice. A total of 331 participants (46.88%) agreed with its statements (*p* < 0.001). "Debt Ratio" had the highest agreement rate (50.71%). The last category, efficiency, focuses on the ability of pharmacists to utilize their assets and manage inventory effectively. A significant portion of the sample, 331 participants (46.88%), agreed with its related statements. All efficiency indicators showed significant mean differences (*p* < 0.05). "Inventory Turnover" had the highest agreement rate (52.41%). The total mean score for all financial performance indicators was 3.30 ± 0.96 (*p* < 0.001), indicating that the financial performance indicators collectively showed a significant difference. This suggests that the financial performance of pharmacists, as evaluated by the various metrics, varies significantly within the sample.

**Table 3:** The analysis of the most relevant pharmacists’ financial performance indicators utilized (N = 353).

No.	Variables Statements	Agreed and Strongly Agreed	Sample’s Mean	Mean Significance	
		N (%)	M ± SD	*t*	*p-*value
**I. Profitability**		747 (52.90)	3.44 ± 0.96	8.72	<0.001
**1**	Profit Margin	238 (67.42)	3.80 ± 1.05	14.41	<0.001
**2**	Gross Profit Margin	201 (56.94)	3.61 ± 1.06	10.76	<0.001
**3**	Return on Asset	172 (48.73)	3.31 ± 1.17	4.98	<0.001
**4**	Return on Equity	136 (38.53)	3.05 ± 1.21	0.79	0.428
**II. Liquidity**		258 (36.54)	3.05 ± 1.15	0.74	0.459
**5**	Current Ratio	129 (36.54)	3.04 ± 1.19	0.63	0.531
**6**	Quick Ratio	129 (36.54)	3.05 ± 1.19	0.81	0.420
**III. Leverage**		331 (46.88)	3.28 ± 1.12	4.70	<0.001
**7**	Debt Ratio	179 (50.71)	3.38 ± 1.14	6.23	<0.001
**8**	Debt to Equity Ratio	152 (43.06)	3.18 ± 1.23	2.76	0.006
**IV. Efficiency**		331 (46.88)	3.29 ± 1.09	4.95	<0.001
**9**	Asset Turnover	146 (41.36)	3.16 ± 1.17	2.58	0.010
**10**	Inventory Turnover	185 (52.41)	3.41 ± 1.14	6.82	<0.001
**Total**		1667 (47.22)	3.30 ± 0.96	5.89	<0.001

Table 4 shows significant differences in pharmacists’ financial perceptions, practices, and use of indicators. The summarized results reveal significant variations in pharmacists’ perceptions, practices, and utilization of financial indicators. Regarding pharmacy ownership, while no significant differences were found, chain pharmacies reported a notably higher use of profitability indicators compared to single pharmacies, with a mean of 3.80 and 3.41, respectively. Regarding the province, pharmacist practices and activities varied significantly, with the middle province scoring higher results (mean of 3.63), and profitability indicators were significantly higher in the north province (mean of 3.53).

**Table 4:** Summary of significant differences in pharmacists’ financial perception, practices, and use of indicators (N = 353).

Dimension	Sub-Dimension	Groups	Significant Differences (*p* < 0.05)
**Ownership Status (Single or Chain Pharmacy) Comparison**			
**Perception and Importance**	**-**	Single vs. Chain Pharmacy	No Differences (*p* = 0.299)
**Financial Indicators Used**	**Profitability**	Single vs. Chain Pharmacy	Yes (*p* = 0.035)
**Province (North, Middle, South) Comparison**			
**Practices and Activities**	**-**	North, Middle, South	Yes (*p* < 0.001)
**Financial Indicators Used**	**Profitability**	North vs. South	Yes (*p* = 0.027)
**Financial Indicators Used**	**Efficiency**	North vs. South	Yes (*p* = 0.015)
**Practices and Activities**	**-**	Middle vs. North, South	Yes (*p* = 0.014, *p* = 0.006)
**Financial Indicators Used**	**Profitability**	Middle vs. South	No Differences (*p* = 0.050)
**Financial Indicators Used**	**Efficiency**	Middle vs. South	Yes (*p* = 0.049)
**Foundation Age (Less than 5 years, 5–9 years, 10 years or older) Comparison**			
**Perception and Importance**	**-**	Less than 5 years, 5–9 years, 10 years or older	No Differences (*p* = 0.854)
**Financial Indicators Used**	**Profitability**	Less than 5 years vs. 10 years or older	Yes (*p* = 0.013)
**Financial Indicators Used**	**Liquidity**	Less than 5 years vs. 10 years or older	Yes (*p* = 0.034)
**Financial Indicators Used**	**Leverage**	Less than 5 years vs. 10 years or older	Yes (*p* = 0.005)
**Financial Indicators Used**	**Liquidity**	Less than 5 years vs. 5–9 years, 10 years or older	Yes (*p* = 0.026, *p* = 0.020)
**Financial Indicators Used**	**Leverage**	Less than 5 years vs. 5–9 years, 10 years or older	Yes (*p* = 0.034, *p* = 0.006)
**Practical Experience (Less than 5 years, 5–9 years, 10 years or older) Comparison**			
**Perception and Importance**	**-**	Less than 5 years, 5–9 years, 10 years or older	Yes (*p* = 0.044)
**Financial Indicators Used**	**Profitability**	Less than 5 years vs. 10 years or older	Yes (*p* = 0.013)
**Financial Indicators Used**	**Liquidity**	Less than 5 years vs. 10 years or older	Yes (*p* = 0.003)
**Financial Indicators Used**	**Leverage**	Less than 5 years vs. 10 years or older	Yes (*p* = 0.005)
**Financial Indicators Used**	**Profitability**	10 years or older vs. less than 5 years, 5–9 years	Yes (*p* = 0.023, *p* = 0.015)
**Financial Indicators Used**	**Liquidity**	10 years or older vs. less than 5 years, 5–9 years	Yes (*p* = 0.001, *p* = 0.001)
**Financial Indicators Used**	**Leverage**	Less than 5 years vs. 5–9 years	Yes (*p* = 0.001)

Additionally, province-based post hoc analysis revealed consistent disparities in practices and activities, profitability, and efficiency, with the middle province outperforming the south province with a mean of 3.51 and 3.05, respectively. Examination of foundation age showed no differences in the perception of financial indicators. However, pharmacies with less than five years of foundation age scored significantly higher in profitability (mean of 3.53), liquidity (mean of 3.29), and leverage (mean of 3.54) indicators compared to those with ten years or more (means of 3.42, 2.96, and 3.16, respectively). Further analysis showed significant differences in liquidity and leverage between groups, with those having less than five years of experience scoring higher.

In contrast, practical experience influenced pharmacists’ financial perception, with those having ten years or more of experience demonstrating significantly higher scores. Further tests underscored significant profitability, liquidity, and leverage indicator differences among different practical experience groups. These factors influence pharmacists’ financial decision-making and can inform tailored interventions and education in the field, potentially enhancing financial practices and overall performance among pharmacists.

## Discussion

The findings of this study highlight the perception of financial indicators among community pharmacists in Jordan, providing essential insights into the financial literacy and attitudes of this vital healthcare sector workforce. The relatively balanced gender distribution is encouraging, as it suggests that the pharmacy profession is attracting individuals from diverse backgrounds, with 53.5% females and 46.5% males. This aligns with previous research emphasizing the diversity and equal opportunities present in the field or any potential implications of empowering the number of women in the profession [30].

The study also demonstrates that a significant proportion of pharmacists were owners (78.8%), and a substantial number had more than ten years of experience (56.7%). The ownership and experience of pharmacists are key factors that can influence financial management practices and potentially contribute to the success and sustainability of their businesses. This underscores the role that experienced pharmacists and pharmacy ownership play in shaping the financial landscape of community pharmacies. Moreover, regarding the diverse range of methods for assessing the financial performance of pharmacies, a majority relied on self-effort based on experience and knowledge (64.6%), while almost one-fourth hired a professional accountant, indicating a variety of approaches to financial management within the profession. Understanding these practices can aid in developing tailored financial management strategies and educational programs for pharmacists.

The results also indicate that a significant proportion of community pharmacists in Jordan are familiar with various financial indicators in use, with the highest levels of awareness observed for profitability and liquidity indicators. This finding is consistent with the role of community pharmacists as healthcare providers and business managers, necessitating familiarity with financial concepts to ensure the sustainability of their pharmacies [31]. This also aligns with the assertion that pharmacists must excel in multiple areas, including financial management, to run their business effectively [32].

The positive perceptions (3.30 ± 0.96) and adequate practice (3.50 ± 0.92) of financial indicators among the surveyed pharmacists are noteworthy. Most participants agreed on the importance of these indicators for managing finances, tracking financial health, and making informed decisions. This alignment with the economic responsibilities outlined in Carroll’s corporate social responsibility pyramid [25] reinforces the idea that pharmacy practice is inherently intertwined with financial performance, especially in the context of community pharmacies that operate as businesses. Financial indicators serve as valuable tools to measure the economic health of these establishments, aligning with a systematic review’s exploration of financial metrics for community pharmacy success in any country [33].

A strong commitment among pharmacists and active engagement in various financial practices and activities were observed. These findings emphasize the importance of effective financial management within the pharmacy profession and its potential impact on healthcare organizations. These practices play a crucial role in ensuring the sustainability and success of pharmacies, which, in turn, contributes to the provision of quality healthcare services to the community, as reported in the literature [18, 34–36]. The high levels of agreement and significance in the responses underscore the robustness of these financial practices. These results contribute to better implications for developing best practices and financial training for pharmacists to enhance their overall management capabilities.

Regarding the utilized financial performance, the findings suggest that pharmacists generally perceive their financial performance positively in terms of profitability (*t* = 8.72), leverage (*t* = 4.70), and efficiency (*t* = 4.95). However, their views on liquidity indicators are more neutral (*t* = 0.74). It is important to note that the significance of these findings varies, with some indicators holding more substantial fluctuation in pharmacists’ perceptions than others. These results provide valuable insights into the financial mindset of pharmacists. They may have implications for their financial decisions, strategies, pharmacists’ financial well-being, and the pharmacy profession as a whole [37]. In addition to these implications, ethical challenges should be considered due to the intersection and influence between pharmacists’ financial decisions and their professional commitment, as revealed in a qualitative study on the ethical challenges in community pharmacy practice [38].

Furthermore, the findings shed light on the complex interplay of factors influencing pharmacists’ financial perceptions, practices, and the utilization of financial indicators in their daily operations. Identifying and addressing these variations to enhance financial decision-making within the pharmaceutical field is essential. Notably, the data revealed that chain pharmacies exhibit a significantly higher utilization of profitability indicators than single pharmacies (*p* = 0.035). While the disparity was evident, it is crucial to investigate the underlying reasons for this discrepancy. Chain pharmacies may have more extensive resources and infrastructure, enabling them to adopt sophisticated financial management practices. Single pharmacies, on the other hand, may face resource constraints, potentially limiting their ability to focus on profitability measures [39]. Recognizing these differences is essential, as it allows for tailored financial education and support, ensuring that single pharmacies can benefit from the financial best practices observed in chain pharmacies.

The significant variations observed across provinces in terms of practices and activities (*p* < 0.001), profitability (*p* = 0.027), efficiency (*p* = 0.015), and overall financial performance (*p* = 0.004) underline the impact of regional context on financial practices. Specifically, the middle province exhibited notably higher scores, suggesting a more favorable financial environment for pharmacists. These show the importance of tailoring financial education and intervention strategies to each province’s needs and challenges. Targeted approaches, accounting for regional differences, can help pharmacists in less favorable regions improve their financial practices and, therefore, their overall financial performance. These potential solutions can inform regional support systems and policies to address disparities and improve financial practices, in line with previous research findings [40, 41].

The analysis of foundation age and practical experience uncovered interesting patterns (*p* < 0.05). Pharmacies under five years of foundation age demonstrated higher scores in profitability, liquidity, and leverage indicators. This suggests that newer entrants into the pharmacy profession may bring fresh perspectives and a more proactive approach to financial management. Their focus on profitability and financial liquidity is particularly notable and aligns with the need for early-career pharmacists to establish sound financial practices. Conversely, pharmacists with ten years or more of practical experience exhibited a notably higher perception of financial indicators. This may indicate a deeper understanding of financial concepts and a more strategic approach to financial management. Their expertise can serve as a valuable resource for mentoring and guiding less experienced pharmacists.

Further analysis illuminated the significance of experience levels, highlighting the differential impact on financial practices and performance. These findings emphasize the need for tailored financial training programs at different stages of a pharmacist’s career [42]. Early-career pharmacists can benefit from targeted education on profitability and liquidity, while experienced pharmacists may benefit from advanced financial strategies and planning.

In conclusion, the study’s findings offer a comprehensive view of the multifaceted factors influencing pharmacists’ financial decision-making. Recognizing and addressing these variations is important for developing targeted interventions and educational programs within the pharmacy profession. Tailored financial education can equip pharmacists with the skills and knowledge needed to make informed financial decisions, ultimately enhancing their financial practices and overall performance in healthcare settings.

### Implications for pharmacy education and practice

The findings of this study hold several implications for pharmacy education and practice in Jordan. First, academic institutions ought to think about adding courses on financial management to their existing curricula, including both basic and advanced topics. By including financial knowledge in pharmacy curricula, graduates will be better equipped to run their pharmacies successfully. Second, practicing pharmacists should have access to continuing education and development programs so they may maintain current industry best practices and financial understanding. These courses might specifically address the financial challenges faced by community pharmacists in Jordan. Finally, pharmacists need practical tools and resources to facilitate financial analysis in their pharmacies. Developing user-friendly financial software or applications tailored to pharmacy needs could empower pharmacists to make data-driven decisions.

### Limitations and future research

This study on financial indicators among community pharmacists in Jordan has limitations, including its cross-sectional design, restricting the establishment of causality, and tracking changes over time. Self-reporting bias may affect findings, and the potential underrepresentation of certain geographic subgroups is acknowledged. Despite these limitations, the study highlights existing utilization with room for improvement, particularly among specific demographics. Future research could involve more extensive sampling and explore financial literacy among pharmacy students. Addressing identified challenges may enhance recognition of financial indicators’ significance, informing educational and professional development initiatives in pharmacy practice.

## Conclusion

This comprehensive study sheds light on the financial literacy, awareness, and utilization of financial indicators among community pharmacists in Jordan. The findings highlight positive perceptions, underscoring the importance of effective financial management within the pharmacy profession. This research has important implications for pharmacy education and practice, suggesting the need for enhanced curricula, continuous education, practical financial tools, and further exploration of the financial literacy of pharmacy students. Addressing these aspects can contribute to improved financial practices and overall performance among community pharmacists in Jordan. Lastly, support the sustainability of pharmacy businesses and the delivery of quality healthcare services.

## Supporting information

S1 Appendix The questionnaire used in this study. (DOCX)

S1 Dataset The study findings raw data. (XLSX)
