# Supplementary material for: Financial indicators utilization among community pharmacists: A comprehensive study for pharmacy management
Source: PLoS One. 2024 Mar 1;19(3):e0299798. doi: 10.1371/journal.pone.0299798 (PMC10906900; doi:10.1371/journal.pone.0299798)
Supplement: S1 Appendix — (DOCX) [file pone.0299798.s001.docx]

**Appendix**

This questionnaire forms part of the research that is being carried out by researchers from the College of Pharmacy at Zarqa University and aims to assess the pharmacists who own community pharmacies in Jordan of the concept of financial performance indicators (indicators of profitability, liquidity, leverage, and efficiency) and identify the financial indicators that they use in evaluating the financial performance of their pharmacies.

We kindly ask you, if you agree to participate in this questionnaire, to answer its interlocutor in light of the practical reality that you are facing, and we assure you that all data and information obtained will be treated confidentially and securely and will be used for scientific research purposes only.

We appreciate and thank you for your participation and cooperation, which contributes to the dissemination of science and knowledge, the exchange of experiences, and helps solve health and societal problems.

Do you agree to participate in this survey?

- Yes, I agree to participate.
- I do not agree to participate.

**Part One: Demographic Information**

Please choose or select the answer that applies to you:

1. **Gender:**
   - Male
   - Female
2. **The age: _______.**
3. **Qualification:**
   - Pharmacist (BSc)
   - Doctor Pharmacy (PharmD)
   - Graduate Studies
4. **The province in which the pharmacy is located:**

- South Province
- Middle Province
- North Province

1. **Pharmacy Ownership:**

- Individual pharmacy
- Chain Pharmacies

1. **The age of pharmacy:**

- Less than five years
- Between 5 to 10 years
- More than ten years

1. **Pharmacist job description:**

- Pharmacy owner
- Financial commissioned pharmacist

1. **Practical experience in community pharmacies:**

- Less than five years
- Between 5 to 10 years
- More than ten years

1. **We obtain financial performance information (indicators of profitability, Liquidity, Leverage, and Efficiency) for the pharmacy through:**

- Professional accountant
- Self-effort, according to our experience and knowledge
- financial accounting Company
- We do not evaluate the financial performance of the pharmacy
- Other (please specify)

1. **The pharmacy uses a special electronic accounting program (software) to process pharmacy accounts and obtain financial reports:**

- Yes, Smart
- Yes, Galaxy
- Yes, Falcon
- Yes, Darwazeh
- Yes, Dawatec
- Yes, other software
- We do not use any accounting software to process pharmacy accounts and obtain financial reports.

**The second part:** the focus of the application of financial performance analysis:

**Part (A):** The focus of the practices and activities of financial performance evaluation by pharmacists who own community pharmacies.

Please put a tick (✔) in the box indicating the degree of agreement against each of the following statements:

| **Variables Statements** | Strongly agree | Agree | Neutral | Disagree | Strongly disagree |
| --- | --- | --- | --- | --- | --- |
| We do a financial analysis of the pharmacy periodically. |  |  |  |  |  |
| We prepare the balance sheet. |  |  |  |  |  |
| We prepare the income statement. |  |  |  |  |  |
| We prepare a cash flow statement |  |  |  |  |  |
| We prepare the trial balance at the end of the financial period. |  |  |  |  |  |
| We compare the primary objectives of the balance sheet and income statement. |  |  |  |  |  |
| We make a relationship between the balance sheet and the income statement for a particular financial year. |  |  |  |  |  |
| We divide the net profit by the total revenue to find the pharmacy's profit margins |  |  |  |  |  |
| We follow up on payables and receivables periodically. |  |  |  |  |  |
| We calculate the amount of change in monthly revenue |  |  |  |  |  |
| We prepare financial statements using Excel |  |  |  |  |  |
| We use computer programs to generate financial statements. |  |  |  |  |  |

**Part (B):** Determining the financial performance indicators that are used by pharmacists who own community pharmacies

Please put a tick (✔) in the box that indicates the extent to which each of the following indicators is used to assess the financial performance of your pharmacy:

| **Variables Statements** | Strongly agree | Agree | Neutral | Disagree | Strongly disagree |
| --- | --- | --- | --- | --- | --- |
| **Profitability Indicators** | | | | | |
| Net profit margin |  |  |  |  |  |
| Gross profit margin |  |  |  |  |  |
| Return on asset |  |  |  |  |  |
| Return on equity |  |  |  |  |  |
| **liquidity indicators** | | | | | |
| Current ratio |  |  |  |  |  |
| Quick ratio |  |  |  |  |  |
| **Leverage Indicators** | | | | | |
| Debt ratio |  |  |  |  |  |
| Debt to equity ratio |  |  |  |  |  |
| **Efficiency Indicators** | | | | | |
| Asset turn over |  |  |  |  |  |
| Inventory turn over |  |  |  |  |  |
